# Persistent Changes of Peripheral Blood Lymphocyte Subsets in Patients with Oral Squamous Cell Carcinoma

**DOI:** 10.3390/healthcare10020342

**Published:** 2022-02-10

**Authors:** Ana Caruntu, Liliana Moraru, Mihaela Surcel, Adriana Munteanu, Daniel Octavian Costache, Cristiana Tanase, Carolina Constantin, Cristian Scheau, Monica Neagu, Constantin Caruntu

**Affiliations:** 1 Department of Oral and Maxillofacial Surgery, The “Carol Davila” Central Military Emergency Hospital, 010825 Bucharest, Romania; ana.caruntu@gmail.com (A.C.); liliana.moraru@yahoo.com (L.M.); 2 Department of Oral and Maxillofacial Surgery, Faculty of Dental Medicine, “Titu Maiorescu” University, 031593 Bucharest, Romania; 3 Immunology Department, Victor Babes National Institute of Pathology, 050096 Bucharest, Romania; msurcel2002@yahoo.com (M.S.); iadinuta@yahoo.com (A.M.); caroconstantin@gmail.com (C.C.); 4 Faculty of Biology, University of Bucharest, 030018 Bucharest, Romania; 5 Department of Dermatology, The “Carol Davila” Central Military Emergency Hospital, 010825 Bucharest, Romania; daniel_costache@yahoo.com; 6 Biochemistry Laboratory, Victor Babes National Institute of Pathology, 050096 Bucharest, Romania; cristianatp@yahoo.com; 7 Faculty of Medicine, “Titu Maiorescu” University, 031593 Bucharest, Romania; 8 Department of Pathology, Colentina University Hospital, 020125 Bucharest, Romania; 9 Department of Physiology, The “Carol Davila” University of Medicine and Pharmacy, 050474 Bucharest, Romania; costin.caruntu@gmail.com; 10 Department of Dermatology, Prof. N.C. Paulescu National Institute of Diabetes, Nutrition and Metabolic Diseases, 011233 Bucharest, Romania

**Keywords:** flow cytometry, head and neck cancer, lymphocyte subpopulations, peripheral circulation, squamous cell carcinoma

## Abstract

Background: Oral squamous cell carcinoma (OSCC) is a common cancer with high morbidity and mortality. Alterations of antitumor immune responses are involved in the development of this malignancy, and investigation of immune changes in the peripheral blood of OSCC patients has aroused the interest of researchers. Methods: In our study, we assessed the proportions of CD3+ total T lymphocytes, CD3+CD4+ helper T lymphocytes, CD3+CD8+ suppressor/cytotoxic T lymphocytes, CD3−CD19+ total B lymphocytes, and CD3−CD16+CD56+ NK cells in the peripheral blood of OSCC patients. Results: The data obtained both pre- and post-therapy showed a similar level of total CD3+ T lymphocytes in OSCC patients and control subjects, pinpointing the stability of this immune parameter. On the other hand, pre-therapeutic data showed a lower proportion of helper T lymphocytes (CD4+), a significantly higher level of cytotoxic/suppressive T lymphocytes (CD8+), and a much lower CD4+ T lymphocyte/CD8+ T lymphocyte ratio compared to control subjects. Conversely, evaluation of circulating NK (CD16+) cells showed a markedly higher pre-therapeutic level compared to the control group. Conclusions: Our results related to immune changes in the peripheral blood add new information to this complex universe of connections between immuno-inflammatory processes and carcinogenesis.

## 1. Introduction

Oral squamous cell carcinoma (OSCC) is a major type of head and neck cancer and one of the most common malignancies [1,2], associated with high morbidity and mortality rates, especially in advanced stages [3].

The multifactorial etiology of the disease, implying both endogenous and environmental factors [4,5,6,7,8,9,10,11,12,13], contributes to the complexity of the mechanisms related to the development of OSCC. Impairment of immune defense mechanisms plays a major role in the onset and progression of neoplastic processes [14,15,16,17,18,19,20,21,22]. Thus, investigation of the distribution and functionality of immune cells in different types of cancer and their connections with the local and systemic disease control has drawn the attention of the scientific world in the last years [23,24,25,26].

Numerous studies have evaluated the peripheral representation of immune cells in different types of neoplasms, and recent research suggests their possible prognostic role [27,28,29].

Lymphocytes are the main immune effectors in head and neck squamous cell carcinoma (HNSCC), particularly with oral localization, and in this pathology, changes in peripheral blood lymphocyte subtypes have been described in both cytotoxic T lymphocytes, helper T lymphocytes, and regulatory T cells, suggesting a link between the development of oral and maxillofacial squamous cell carcinoma (SCC) and the alteration of the antitumor immune responses [30,31,32]. Along with T lymphocytes, natural killer (NK) cells play a major role in antitumor defense, and alterations of number, proportion, and function of circulating NK cells have been reported in oral squamous cell carcinoma [33,34]. Although the changes of B lymphocytes in oral and maxillofacial SCC have not been extensively investigated, studies suggest important modulatory actions associated with B cells [35].

All the gathered data support the major role of lymphocytes in the pathogenesis of OSCC. In-depth analysis of the effects of different lymphocyte subtypes could contribute to a better understanding of immune processes and their correlations with carcinogenesis.

In this study, we aimed to investigate the proportion of lymphocyte subtypes in the peripheral blood of OSCC patients, before treatment and after its completion, to evaluate the potential impact of therapy on the immune mechanisms associated with this pathology.

## 2. Materials and Methods

### 2.1. Study Protocol

This observational study included patients with a histopathological confirmed diagnosis of OSCC, treated in the Department of Oral and Maxillofacial Surgery, “Carol Davila” Central Military Emergency Hospital, Bucharest. The study was conducted in accordance with the Declaration of Helsinki (1964), with the approval of the Local Ethics Committee (No. 25/27 November 2017), and each patient and control subject included in the study was informed of the study protocol and signed the informed consent form.

The study included 13 patients with operable forms of OSCC and a control group of 20 subjects without neoplastic, infectious, or inflammatory pathology and with demographic characteristics comparable to the OSCC group (age and gender).

All patients in OSCC group met the following inclusion criteria: diagnosis of OSCC with histopathological confirmation, in operable stages with curative visa, and having no previous treatments (surgical, chemotherapy, radiotherapy, immunotherapy). Patients with other malignancies, immunological conditions, or other severe, decompensated conditions were excluded. The clinical examination was completed with imaging investigations (CT/MRI) for optimal staging and treatment planning.

During the preoperative preparations, the patients were evaluated both biologically and cardio-pulmonary. Biological evaluation involved, in addition to the usual investigations, the collection of peripheral blood samples to determine circulating lymphocyte subtypes using the flow cytometry technique.

The surgical treatment consisted in the excision of the tumor, with oncological safety limits and the corresponding reconstruction of the defect (primary/by local or freely vascularized flaps). For some patients, in the same surgical session or in different sessions, prophylactic or curative lymph node dissection was performed, depending on the lymph node status established preoperatively. Patients then underwent adjuvant radiotherapy with/without sensitization chemotherapy and/or active dispensing, according to national therapeutic guidelines. The adjuvant treatment schedule was established within the oncology commission in the territorial centers where the patients were treated. Three months after the end of the surgical and/or adjuvant treatment, the patients returned to the oncology follow-up program, at which point, a second peripheral blood sample was collected to evaluate post-therapeutic changes in circulating lymphocyte subtypes. Of the 13 OSCC patients included in the study, the second collection session could not be performed for 2 patients. Patients followed an active follow-up program for 5 years after surgical treatment.

### 2.2. Flow Cytometry Analysis

The lymphocytes immunophenotyping was performed from EDTA-anticoagulated whole peripheral blood by flow cytometry, based on the expression of cellular surface markers. We used a BD Multitest IMK Kit (IVD) (Becton, Dickinson and Company, Franklin Lakes, NJ, USA). The kit contains two reagents (Multitest 1 and Multitest 2) and lysis solution 10X concentrated. Each reagent is a mixture of four monoclonal antibodies conjugated with different fluorochromes: CD3-Fluorescein Isothiocyanate (FITC)/CD8-Phycoerythrin, (PE)/CD45-Peridin-Chlorophyll-Protein, (PerCP)/CD4-Allophycocyanin (APC), and CD3-FITC/CD16+CD56-PE/CD45-PerCP/CD19-APC.

The staining protocol was performed according to producers: for each sample, the whole blood was incubated with Multitest 1 and Multitest 2 reagents for 15 min at room temperature and in the dark. Surface staining was followed by red blood cell lysis with lysing solution 1X (supplied by the kit) for 15 min at room temperature and in the dark and was then analyzed by flow cytometry. Data acquisition and analysis were performed on a BD FACSCanto II cytometer with BD FACSCanto clinical software (Becton, Dickinson and Company, Franklin Lakes, NJ, USA). Cytometer performances were checked using 7 Color Setup Beads (Becton, Dickinson and Company, Franklin Lakes, NJ, USA).

The quantified lymphocyte populations and subpopulations were as follows: T-CD3+ lymphocytes (CD45+CD3+) with T-CD4+ (CD45+CD3+CD4+) and T-CD8+ (CD45+CD3+CD8+) subsets, B lymphocytes (CD45+CD3−CD19+), and NK cells (CD45+CD3−CD16+CD56+).

The clinical program BD FACSCanto uses a working document imposed by the manufacturer and finally provides a report (Lab Report) with dot-plots and results. Gating strategy contains four dot-plots. The first two dot-plots are common for both tubes, namely CD45 PerCP vs. SSC-A and CD3 FITC vs. SSC-A. Lymphocyte population is selected using CD45 PerCP vs. SSC-A dot-plot, and based on the presence or absence of CD3, from the second dot-plot (CD3 FITC vs. SSC-A), lymphocytes CD3+ and CD3− are selected. For the first tube, from lymphocytes CD3+ population (total T lymphocytes), there were identified helper T lymphocytes (CD3+CD4+) and suppressor/cytotoxic T lymphocytes (CD3+CD8+), using a quadrant applied in the CD8 PE vs. CD4 APC dot-plot. For the second tube, from lymphocytes CD3- population, there were identified B lymphocytes (CD3−CD19+) and NK cells (CD3−CD16+CD56+), using a quadrant applied in the CD16+56 PE vs. CD19 APC dot-plot.

### 2.3. Statistical Analysis

For the statistical analysis, we used Prism 9 software (GraphPad Software, San Diego, CA, USA). We evaluated the normality of the data distribution using the Kolmogorov–Smirnov test. We used the Fisher test to analyze the gender distribution and the *t*-test to compare the age of the subjects in the two study groups. To investigate the proportion of lymphocyte subtypes in peripheral blood before and after treatment, we used the paired *t*-test or the Wilcoxon test if the data did not have a normal distribution. To evaluate the differences between the data of patients with OSCC and the subjects in the control group, we used the *t*-test or the Mann–Whitney test if the data did not have a normal distribution. The results are presented as mean ± standard deviation (SD); *p*-values < 0.05 were considered statistically significant.

## 3. Results

### 3.1. General Characteristics of the Study Groups

The general characteristics of OSCC patients and those of control subjects are presented in Table 1. We chose control subjects with the same age range acknowledging that age per se alters the lymphocyte’s subpopulation proportion [36]. The clinical and histopathological details of OSCC patients are presented in Table 2.

### 3.2. Evaluation of Circulating Lymphocyte Subtypes before and after Treatment

The analysis performed did not reveal any statistically significant difference between pre- and post-therapeutic levels for any of the studied lymphocyte populations studied (Table 3 and Table 4 and Supplementary Figure S1). This result strengthens the constancy of certain cellular immune parameters in OSCC patients and the fact that tumor onset is sustained by an immune deregulation.

### 3.3. Comparative Investigation of Peripheral Blood Lymphocyte Subtypes in OSCC Patients and Control Subjects

Analysis of circulating lymphocyte subtypes in patients with OSCC evaluated pre- and post-therapeutically showed significant differences compared to the control group (Table 3 and Table 4 and Supplementary Figure S1).

Regarding the level of total T lymphocytes (CD3+), no statistically significant differences were noticed in either the pre-therapy or post-therapy OSCC groups compared to the control subjects. However, we highlighted important changes regarding the various lymphocyte subtypes.

Thus, in OSCC patients, the pre-therapy level of helper T lymphocytes (CD4+) was significantly lower than the control group (*p* = 0.0018), and the difference was maintained after therapy (*p* = 0.0019).

In contrast, the level of cytotoxic/suppressive T lymphocytes (CD8+) was significantly higher both pre- and post-therapeutic compared to control subjects (*p* < 0.0001).

These changes are also significant by analyzing the ratio of CD4+ T lymphocytes/CD8+ T lymphocytes, which was much lower in OSCC patients both before and after treatment.

Furthermore, the level of B lymphocytes (CD19+) was significantly reduced in the group of OSCC patients both pre- and post-therapy.

On the other hand, the pre-therapeutic level of NK cells (CD16+) was markedly increased, but after the end of the treatment, no significant difference could be highlighted compared to the control subjects.

## 4. Discussion

The study of immune changes in the peripheral blood of oral and maxillofacial SCC patients has risen the interest of researchers both for a better understanding of the pathophysiological mechanisms involved in this type of pathology and for the identification of new possible biomarkers with clinical impact.

In our study, we investigated the proportions of CD3+ total T lymphocytes, CD3+ CD4+ helper T lymphocytes, CD3+ CD8+ suppressor/cytotoxic T lymphocytes, CD3-CD19+ total B lymphocytes, and CD3-CD16+ CD56+ NK cells in the peripheral circulation of OSCC patients.

The cellular parameters evaluation was performed in dynamics, pre-, and post-therapeutic and compared to control subjects with similar demographic characteristics.

Regarding the comparative investigation of circulating lymphocyte subtypes between groups, the data obtained both pre- and post-therapy showed a similar level of total CD3+ T lymphocytes in OSCC patients and control subjects, pinpointing the stability of this immune parameter. On the other hand, pre-therapeutic data showed a lower proportion of helper T lymphocytes (CD4+), a significantly higher level of cytotoxic/suppressive T lymphocytes (CD8+), and a much lower CD4+ T lymphocyte/CD8+ T lymphocyte ratio compared to control subjects. These differences remained significant even three months after the end of therapy. The immune pattern of the OSCC patients has a possible suppressive feature suggested by the long-term reduction of CD4+ subpopulation.

We acknowledge the limitations of our study regarding the relatively reduced number of investigated patients. However, in our research, the rigorous selection of patients ensured the homogeneity of the study group as regards the tumor location, allowing the evaluation of the lymphocyte subsets particularly in OSCC, this major type of head and neck cancer. The results of our study, including a dynamic investigation before and after therapy and a comparative evaluation with control subjects, bring forward additional information to this complex universe of connections between immuno-inflammatory processes and carcinogenesis, in which the scientific literature still provides an inconclusive picture with some apparently contradictory results.

A recent study evaluating changes in lymphocyte populations in patients with oral and maxillofacial neoplasms versus healthy subjects found similar results to our study in the preoperative percentage of CD3+ T lymphocytes, which was not significantly different from the control group [37]. Another research investigated changes in circulating T lymphocytes associated with oral and maxillofacial SCC [38]. Similar to our study, no significant differences were found between the pre-therapeutic proportions of lymphocytes compared to healthy subjects. In contrast, the level of T lymphocytes was significantly reduced, compared to the control group, in the treated patients, without recurrences.

The results of other studies have shown that both the absolute level and the proportion of T lymphocytes are significantly lower in patients with oral and maxillofacial SCC compared to control [39].

The different results of the studies could be explained by the variability of the groups of investigated patients, as we noticed an inverse correlation between the proportion of T lymphocytes in the peripheral blood and the stage of the disease [40].

Concerning the distribution of T lymphocyte subtypes, a recent study performed in patients with oral and maxillofacial SCC showed results similar to our research. In patients treated for recurrent disease, the percentage of CD4+ lymphocytes was reduced, and that of CD8+ lymphocytes was increased compared to control subjects, with the CD4+/CD8+ ratio being significantly low. On the other hand, in this study, elevated levels of CD3+ and CD4+ lymphocytes were identified in patients with lymph node metastases [41].

A recent research has particularly caught our attention [42]. In their very well-designed multicenter prospective study, Niu et al. investigated the composition of lymphocyte subpopulations in non-locoregional recurrent and locoregional recurrent HNSCC patients. The evaluation was performed before initiation of radiochemotherapy (RCT), after the treatment, and in the follow-up period, and the results were compared with a control group.

In non-relapse patients, the levels of CD3+ T lymphocytes and CD4+ helper T lymphocytes were decreased at each time point of the study period, while the proportions of CD8+ cytotoxic T lymphocytes showed an increasing trend. A similar evolution of T lymphocyte subtypes proportions was emphasized in the locoregional recurrent patients group.

This study also investigated the proportions of CD4+/CD25+/FoxP3+ regulatory T cells (Tregs), which showed an increasing trend in the follow-up period after a first decrease was revealed after application of RCT.

The results of other studies also reported similar changes, with decreased CD4+ helper T lymphocytes, increased CD8+ cytotoxic T lymphocytes, and decreased CD4+/CD8+ ratio in oral and maxillofacial SCC patients compared to control subjects [43]. Moreover, the low percentage of CD4+ T lymphocytes in peripheral blood has been associated with an increased level of CD4+ CD25+ regulatory T cells that may induce an inhibition of antitumor immune responses [44,45,46]. In addition, in the peripheral circulation of patients with oral cancer, a pronounced CD4+ T lymphocyte exhaustion was noticed, especially in the advanced stages of the disease [31]. However, in oral cancer, various studies associated an increased Treg tumor infiltration with a better patient prognosis, showing that, by an increased expression of anti-inflammatory and immunosuppressive cytokines, the Tregs in tumor microenvironment could have a complex and not yet established impact on the tumor-promoting mechanisms [47,48].

Other investigations regarding the percentages of T lymphocyte subsets in the peripheral blood of oral and maxillofacial SCC patients did not show any differences compared to healthy subjects [49,50]. However, analyzing their absolute values, a significant reduction in CD3+, CD4+, and CD8+ T lymphocytes was observed in various studies [49,51].

Other similar studies have indicated different aspects than those obtained in our research. Thus, an increased ratio between helper T lymphocytes and suppressor/cytotoxic T lymphocytes was observed in oral and maxillofacial SCC patients compared to healthy subjects [52]. These divergent results could be explained by the different location of the tumors. In the study performed by Wolf et al., the research included mainly patients with laryngeal tumors alongside patients with oral or maxillofacial involvement. Moreover, the vast majority of patients in this study underwent radiation therapy, which may have a major impact on lymphocyte subpopulations level [52].

Regarding the level of B lymphocytes (CD19+) in the peripheral blood of OSCC patients, our study revealed that their proportion was significantly lower compared to the control group, both pre- and post-therapy.

Niu et al. also emphasized a decreased level of CD19+ B cells before and after application of RCT. Their proportion rose back to the level of healthy subjects only after 6 months of follow-up [42].

Other recent research that analyzed the distribution and functionality of B lymphocyte subsets in oral and maxillofacial SCC did not show significant differences in the peripheral blood of patients compared to control subjects [53]. However, the study suggests the involvement of different B lymphocyte subpopulations in antitumor activity or, vice versa, in promoting tumor development, emphasizing the importance of developing studies focused on peripheral B-cell populations.

In our study, the analysis of circulating NK (CD16+) cells showed a markedly higher pre-therapeutic level compared to the control group. In contrast, the level measured after completion of treatment was not significantly different from the control subjects.

Data provided by other research in patients with oral SCC are mainly focused on changes in the absolute number of circulating NK cells and have shown its decrease and alteration of cytotoxic functions of NK cells [33,51]. They also showed an increase in the number of NK cells in the peripheral circulation after tumor excision [51].

On the other hand, another study on the distribution of immune cells in oral and-maxillofacial SCC evaluated patients before treatment and patients treated without recurrences, compared them to a control group, and showed an increase in the proportion of NK cells in the peripheral circulation of treated patients both compared to untreated patients and to healthy subjects [38].

In the study of Niu et al., in non-relapse patients, the proportions of NK-like T cells (NKT) and NKT/NK cells subsets showed an increasing trend from the first time point, before initiation of RCT, until the end of the study period. However, in patients with locoregional recurrences, a similar trend could not be demonstrated [42].

Our analysis also included a dynamic investigation, which showed similar proportions of circulating lymphocyte subtypes before treatment and three months after its completion, suggesting that any post-therapeutic changes require a longer period of surveillance to become evident. Moreover, the impairment of antitumor lymphocyte mechanisms demonstrated in patients with OSCC may persist for a long time after initiation of therapy [49].

Data from other studies do not provide a convergence of results, and the discrepancies can be explained by the methodological variability and the differences between the groups of evaluated patients.

Regarding the investigation of T lymphocytes, a study that compared their level in the peripheral blood of oral and maxillofacial SCC patients before and after treatment showed an increase in T lymphocytes compared to the initial value [39]. Another investigation, which compared the percentage of T lymphocytes in patients with oral and maxillofacial SCC before treatment and patients treated without recurrences, could not reveal any significant difference between groups [38].

In non-relapse HNSCC patients Niu et al. showed a decreased proportion of CD3+ T lymphocytes after application of RCT, a level that remained low until the end of the study period, after 6-month follow-up [42].

Results of other studies comparing pre- and post-therapeutic lymphocyte subpopulations are also divergent. Thus, recent work showed that tumor resection in patients with oral and maxillofacial cancer is followed by a decrease in CD3+ CD4+ T lymphocytes and an increase in CD3+ CD8+ T lymphocytes [37].

The study of Niu et al. revealed that, in non-relapse HNSCC patients, after application of RCT, the proportion of CD4+ helper T lymphocytes decreases, while the proportions of CD8+ cytotoxic T lymphocytes increases, which are changes that are still present after 6-month follow-up. A similar trend was also noticed in patients with locoregional recurrences.

As we have emphasized, a detailed analysis of these parameters must also take into account the impact of other variables, such as staging and clinical progression.

Although some studies did not show differences regarding lymphocyte populations between patients with recurrent disease and those without recurrences after treatment [54,55,56], other research found different values depending on the stage of the disease. Thus, in the peripheral circulation of patients with oral and maxillofacial cancer in more advanced stages, a lower percentage of T lymphocytes was noticed. However, no prognostic value, additional to clinical staging, could be highlighted [40].

A prospective study found a higher ratio between helper T lymphocytes and suppressor/cytotoxic T lymphocytes in patients with HNSCC in advanced stages of the disease. The study also highlighted the predictive impact of this ratio, an increased value being associated with a poor prognosis [52]. These results are supported by another research that showed correlations of this ratio with the clinical staging and disease progression, suggesting its possible utility as prognostic marker [43].

However, in a recent study of our research group, we revealed significant differences between the tumor tissue and peripheral blood proportions of cytotoxic T cells, helper T lymphocytes, B lymphocytes, and NK cells of untreated HNSCC patients [19]. Thus, the changes in immune cell proportions in peripheral circulation do not necessarily reflect the relationships between immune cells within the tumor microenvironment, and future studies are needed to define their roles as biomarkers in HNSCC.

## 5. Conclusions

The proportions of lymphocyte subtypes in the peripheral blood of OSCC patients included in our study are profoundly altered compared to control subjects, and these changes persist for a long time after the end of treatment. The level of circulatory total T lymphocytes is a stable, un-altered parameter, whereas T-helper lymphocytes are reduced prior to therapy in correlation with an increased T cytotoxic subpopulation. The level of B lymphocytes in peripheral blood is reduced, and as a compensatory immune mechanism, the proportion of NK cells is increased.

Further studies in this area of research will provide a better understanding of the connections between immuno-inflammatory processes and carcinogenesis and will allow the identification of new predictive markers in OSCC.

## Figures and Tables

**Table 1:** General characteristics of the study groups.

Study Group	Gender		Age	
	Male	Female	Mean	SD
OSCC	10	3	67.92	14.96
Control	14	6	71.65	10.74
*p*	>0.999 ^#^		0.4107 ^¶^	

^#^ Fisher test; ^¶^ *t*-test. OSCC, oral squamous cell carcinoma; SD, standard deviation.

**Table 2:** Clinical and histopathological details of OSCC patients.

Patient#	Gender	Age	Tumor Location	Clinical Staging	Histological Differentiation	Treatment
1	F	86	lip	T1N0M0	WD	excision
2	F	85	lip	T2N0M0	MD	excision
3	F	54	tongue	T2N0M0	MD	excision-RT
4	M	88	floor of the mouth	T2N0M0	MD	excision-RT
5	M	80	buccal mucosa	T2N0M0	WD	excision-RT
6	M	80	lip	T1N0M0	WD	excision
7	M	71	gingiva	T4aN1M0	MD	excision-RT
8	M	67	pelvilingual	T3N2M0	MD	excision-RT-sensCHT
9	M	65	gingiva	T2N0M0	MD	excision
10	M	60	retromolar mucosa	T3N2M0	PD	excision-RT-sensCHT
11	M	51	lip	T1N2M0	MD	excision-RT
12	M	51	tongue	T2N2M0	WD	excision-RT
13	M	45	buccal mucosa	T3N1M0	MD	excision

M, male; F, female; WD, well differentiated; MD, moderately differentiated; PD, poorly differentiated; RT, radiotherapy; sensCHT, sensitization chemotherapy; #, number.

**Table 3:** Proportion of lymphocyte subtypes in the three study groups.

Variable (%)	OSCC Pre-Therapy		OSCC Post-Therapy		Control	
	Mean	SD	Mean	SD	Mean	SD
T total	69.461	7.644	69.545	9.554	70.500	5.978
T-CD4+	39.384	9.376	37.091	11.726	50.350	5.612
T-CD8+	28.231	7.617	30.182	9.786	18.100	4.865
T-CD4+/T-CD8+	1.572	0.737	1.429	0.766	3.011	0.955
B	9.923	4.974	9.000	5.639	14.000	2.982
NK	19.769	7.949	20.636	12.363	15.950	3.043

OSCC, oral squamous cell carcinoma; SD, standard deviation; NK, natural killer cells; T, T lymphocytes; B, B lymphocytes.

**Table 4:** Comparative evaluation between groups.

Variable	OSCC Pre-Therapy vs. OSCC Post-Therapy		OSCC Pre-Therapy vs. Control		OSCC Post-Therapy vs. Control	
	*p*	Test	*p*	Test	*p*	Test
T total	0.6409	^#^	0.6653	*	0.7339	*
T-CD4+	0.2152	^#^	0.0018	^^^	0.0019	^^^
T-CD8+	0.1224	^#^	<0.0001	*	<0.0001	*
T-CD4+/T-CD8+	0.0707	^#^	<0.0001	*	<0.0001	*
B	0.0892	^&^	0.0005	^^^	<0.0001	*
NK	0.5833	^#^	0.0118	^^^	0.384	^^^

^#^ paired *t*-test; ^&^ Wilcoxon test; * unpaired *t*-test; ^^^ Mann–Whitney test. NK, natural killer cells; T, T lymphocytes; B, B lymphocytes.

## Data Availability

The datasets generated during and/or analyzed during the current study are available from the corresponding authors on reasonable request.

## Supplementary Materials

The following supporting information can be downloaded at: https://www.mdpi.com/article/10.3390/healthcare10020342/s1, Figure S1: Circulating lymphocyte subtypes in pre- and post-therapeutic OSCC patients and the control group.
