# Supplementary material for: Persistent Changes of Peripheral Blood Lymphocyte Subsets in Patients with Oral Squamous Cell Carcinoma
Source: Healthcare (Basel). 2022 Feb 10;10(2):342. doi: 10.3390/healthcare10020342 (PMC8872623; doi:10.3390/healthcare10020342)
Supplement: Supplementary file 1 [file healthcare-10-00342-s001.zip › healthcare-1569655-supplementary.pdf]

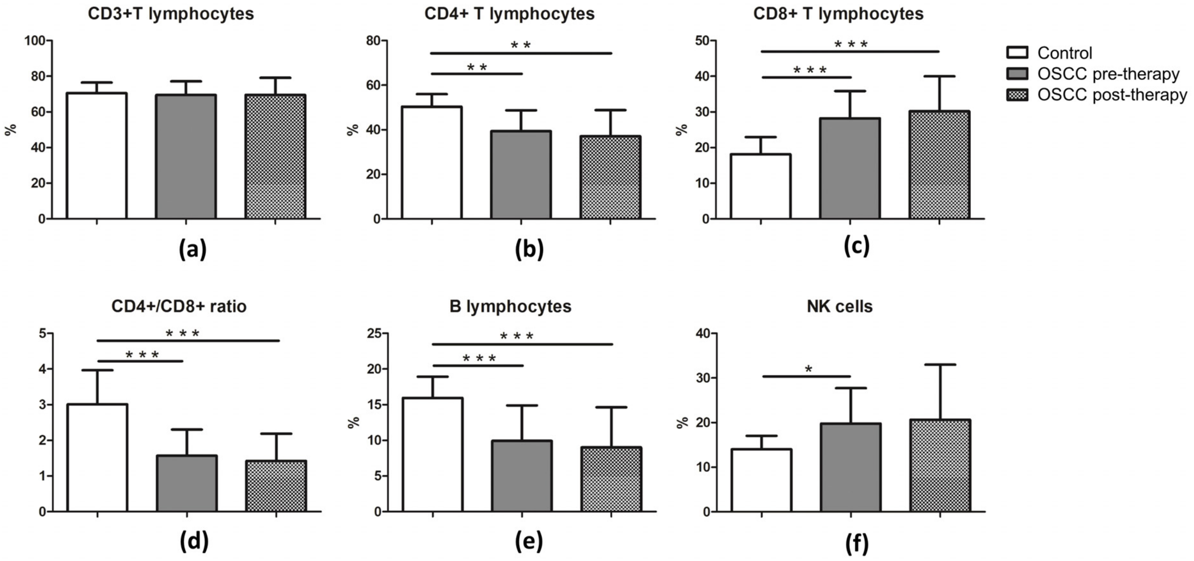

Figure S1: Circulating lymphocyte subtypes in pre- and post-therapeutic OSCC patients and the control group. (a) Proportion of circulating CD3+ T lymphocytes; (b) Percentage of circulating CD4+ T; (c) Proportion of circulating CD8+ T lymphocytes; (d) CD4+ / CD8+ ratio; (e) Proportion of circulating CD19+ B lymphocytes; (f) Proportion of circulating NK CD16+ cells. The error bars represent the standard deviation; * $p < 0.05$, ** $p < 0.01$ and *** $p < 0.001$. OSCC, oral squamous cell carcinoma; CD, cluster of differentiation; NK, natural killer.
